# Lower Lip Reconstruction: Karapandzic Flap

**Published:** 2013-01-23

**Authors:** Aditya Sood, Angie Paik, Edward Lee

**Affiliations:** Division of Plastic Surgery, University of Medicine and Dentistry of New Jersey, Newark

**Figure:**
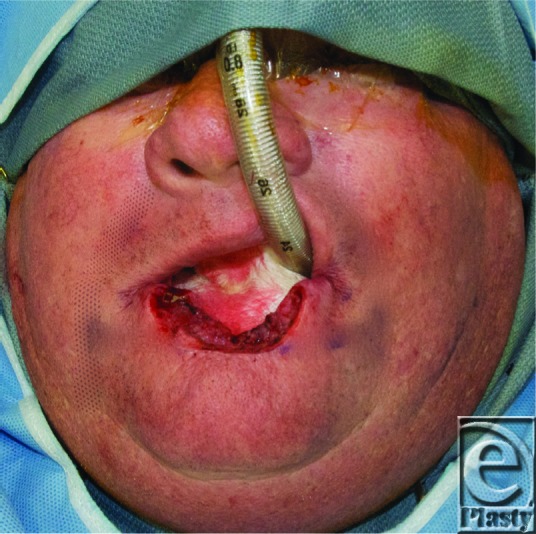


## DESCRIPTION

The patient is a 61 year old man with a shave biopsy proven squamous cell carcinoma (SCC) of the lower lip. Seven years ago, the patient underwent Moh's surgery for the same diagnosis on the lower lip. Patient reports that over the past 6 years the lesion has increased in size, has bled at intervals, and was not healing.

## QUESTIONS

1. **What are the main causes of defects of the lower lip?**
2. **What are risk factors for SCC of the lower lip?**
3. **What considerations must be made for lower lip reconstruction?**
4. **What are the options for lower lip reconstruction?**
5. **What are the advantages and disadvantages of the Karapandzic flap?**

## DISCUSSION

Oncologic resection of cancer remains a major cause of large lip defects. Other causes include trauma, burns, infectious diseases, hemangiomas, and congenital clefts.^1^ Defects of the lower lip are more frequently encountered because of the high incidence of SCC of the lower lip. Squamous cell carcinoma accounts for 95% of lip malignancies with 90% of cases affecting the lower lip, whereas basal cell carcinoma preferentially affects the upper lip.^2^

Unlike other SCC of the head and neck, ultraviolet rays from chronic sun exposure are thought to be a leading cause. Other risk factors include previous radiotherapy and increased age. The management of SCC of the lip depends on extent of spread but is usually excised with possible postoperative radiation for advanced lesions.

The goals of lip reconstruction include oral competence, speech, and cosmesis.^3^ For oral competence, muscular integrity and oral aperture must be preserved. A reduction to less than 50% of stoma size produces oral dysfunction, especially for those who wear dentures. For cosmesis, considerations include maintaining anatomic landmarks as well as aesthetic proportions and symmetry.

In evaluating a lower lip defect, several considerations must be made including the size of the defect, availability of adjacent tissue, and involvement of the commissures. In an algorithm outlined by Aucher et al size of the defect can be broken down into up to one third of the lip, one third to two thirds of the lip, and more than two thirds of the lip.^4^ Defects of up to one third of the lower lip may be closed primarily. Defects between one third and two thirds of the lip with sufficient lip tissue and commissure involvement may be closed with the Karapandzic (first choice) and the Estlander (second choice). When the commissure is not involved, the defect may be closed with the Karapandzic or the Abbe. Defects of more than two thirds of the lower lip with sufficient adjacent cheek tissue may be closed with the Karapandzic for defects up to 80% or the Bernard-Burow. When there is insufficient adjacent cheek tissue, a free flap may be used to close the defect.

**Figure 1:**
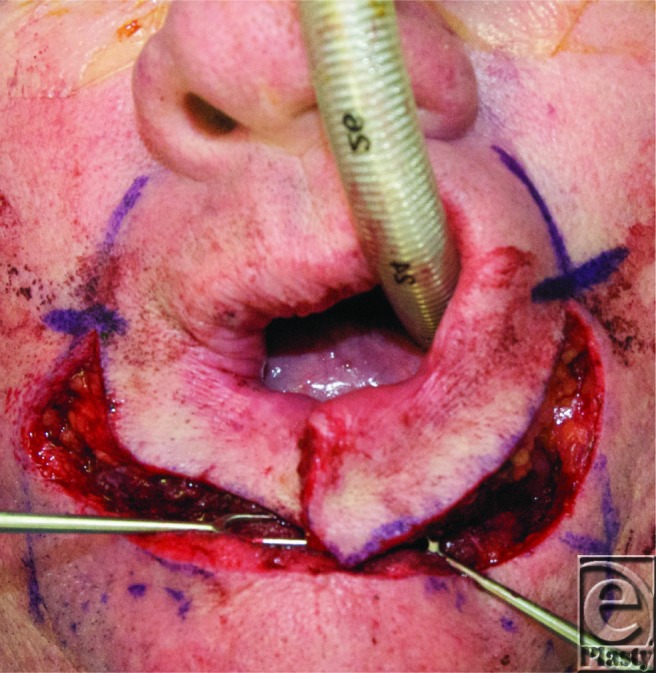
Perioperative: Curvilinear incision leading to alar base with flaps raised.

The Karapandzic flap is created by making a curvilinear incision toward the alar base with the width of the flap equal to the height of the defect (Fig 1). During dissection, the labial arteries and buccal motor nerve branches are identified and preserved and the flap is rotated and advanced for closure.

The advantages of this method are that the flap is fully innervated, so there is preservation of sensation and motor function, and it can be used to seal large defects with similar, adjacent tissue.^5^ Another advantage is that it is a one-stage procedure. The disadvantages are that the lip circumference is reduced and can lead to microstomia and there is rounding or distortion of the commissures. In a prospective case series study with 9 patients with a central defect of 50% to 80%, a 1-year follow-up revealed an overall 70% satisfaction rate with 3 patients who experienced postoperative microstomia that had functional compromise.^2^ These patients went on to have commissuroplasty to correct the microstomia.

Alternative solutions to similar defects have been introduced. In 6 patients who received a 2-stage double-reversed Abbe flap, there was a 90% satisfaction rate, no functional complaints at 3-month follow-up, and no problems with asymmetry or loss of sensation.^2^ In 2011, an extended Karapandzic flap technique was described in patients with larger defects as an alternative to microvascular free flaps and regular Karapandzic flaps with good results.^5^

**Figure 2:**
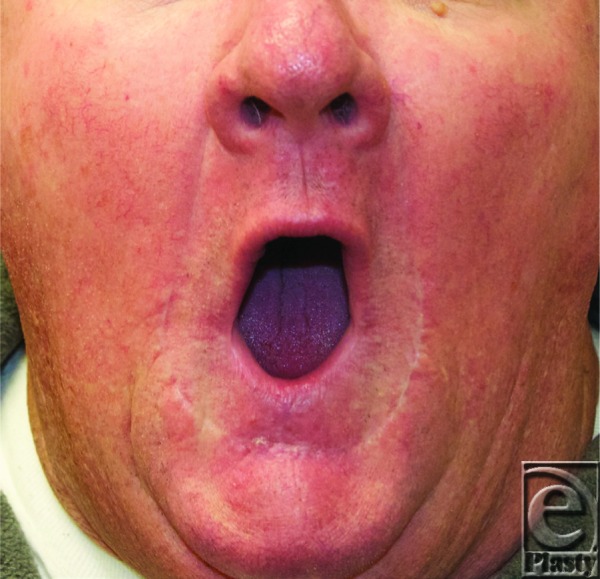
Eight weeks postoperative: Patient demonstrated complete oral competency and became able to wear his full dentures.

In the case described earlier, the patient demonstrated a defect of approximately 80% of lower lip and opted for surgical correction via Karapandzic flap. Eight weeks after operation, the patient demonstrated oral competency, was able to wear his full dentures, and was very happy with the results (Fig 2).
